# Success in the use of oral propranolol in the treatment of infantile hemangioma in nasal tip – Report of two cases^☆^^☆☆^

**DOI:** 10.1016/j.abd.2019.05.005

**Published:** 2020-01-21

**Authors:** Mariana Carvalho Costa, Odil Garrido Campos de Andrade, Lethícia de Castro Pereira, Izelda Maria Carvalho Costa

**Affiliations:** a Service of Dermatology, Hospital Universitário de Brasília, Brasília, DF, Brazil; b Faculdade de Ciências da Saúde e Educação, Centro Universitário de Brasília, Brasília, DF, Brazil

**Keywords:** Hemangioma, Infant, Propranolol

## Abstract

Infantile hemangioma is the most common pediatric vascular tumor, with the following risk factors: low birth weight, prematurity, white skin, female gender, multiparity and advanced maternal age. The use of oral and topical beta-blockers, although recent, has emerged as the first line of treatment, with superior safety and efficacy to previously used therapies, such as corticosteroids and surgeries. This report describes two cases of nasal tip infantile hemangioma, treated with oral propranolol. Both presented excellent therapeutic responses.

## Introduction

Infantile Hemangioma (IH) is the most common pediatric vascular tumor, affecting mainly the nasal tip and head, with incidence of 3% to 10%. Its pathogenesis has yet to be clarified. Relevant risk factors include low birth weight, prematurity, white skin, female gender,^1^, ^2^, ^3^ multiparity and advanced maternal age.^1^, ^2^, ^3^

The clinical course of IH is divided into three phases. First, the rapid phase is characterized by excessive proliferation shortly after birth. Ulcerations, bleeding and obstruction of vital structures can also occur during this phase, especially in preterm infants. Second, the spontaneous regression phase – complete or partial – involves a higher risk of deformities and ulceration. Finally, the plateau phase (in which there is no regression or evolution) arises in approximately 10% of cases, requiring therapeutic intervention, as there is a risk of permanent deformation.^1^, ^2^, ^3^

This report describes two cases of nasal tip IH treated with oral propranolol. Both showed excellent therapeutic responses.

## Case report

**Figure 1:**
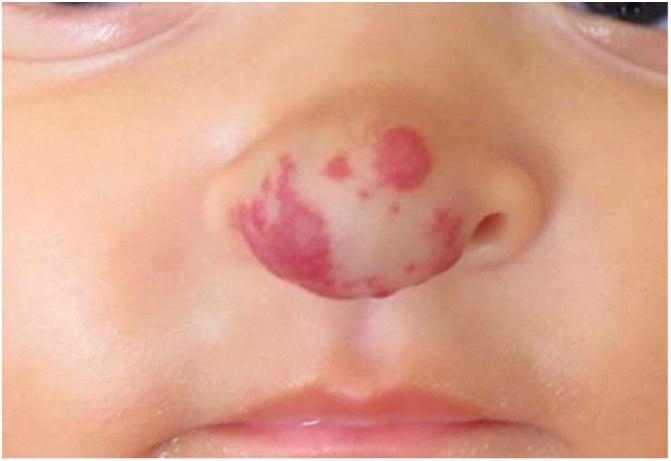
Before the treatment with oral propranolol, 6-month-old infant.

Six-month-old infant, female, presented an erythematous violaceous vascular lesion with increased local volume at the nose tip, compatible with IH (Fig. 1). The lesion appeared in the first days of life as a slightly erythematous macule and manifested progressive enlargement. Born Appropriate for Gestational Age (AGA), full-term infant, multiparous mother, without complications during pregnancy or impairments to psychomotor development.

**Figure 2:**
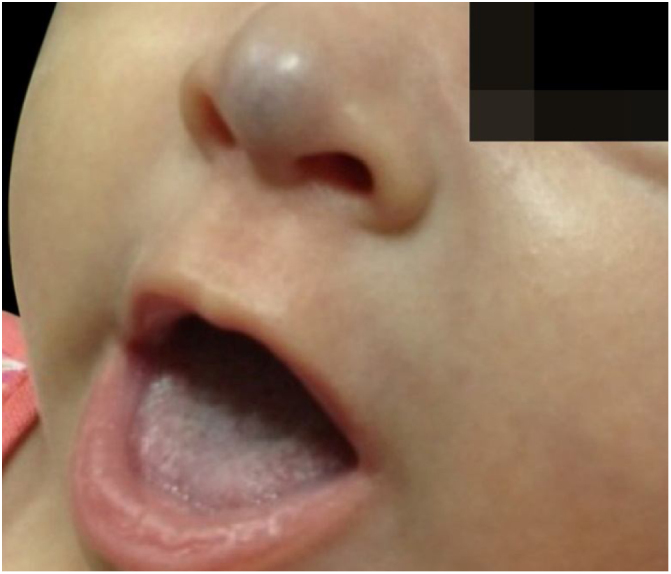
Before the treatment with oral propranolol, 2-month-old infant.

Two-month-old infant, female, presented a violaceous vascular lesion at the nasal tip, surpassing the anatomical limits, compatible with IH (Fig. 2). The lesion appeared in the first days of life as a slightly erythematous and violaceous macule and manifested progressive enlargement. AGA, preterm birth (36 weeks), primiparous mother, in vitro fertilization, with threat of placental abruption and interruption due to oligodramnium, without changes in neuropsychomotor development.

In both cases, patients underwent cardiologic, pulmonary and laboratorial tests, which did not reveal any alterations. In relation to the therapy, the target dose of propranolol was 2 mg/kg/day, bid during meals (first 2 weeks with 0.5 mg/kg/day and a fortnightly progressive increase) over 12 months. The patients were carefully monitored before treatment and when the drug dose was increased.

**Figure 3:**
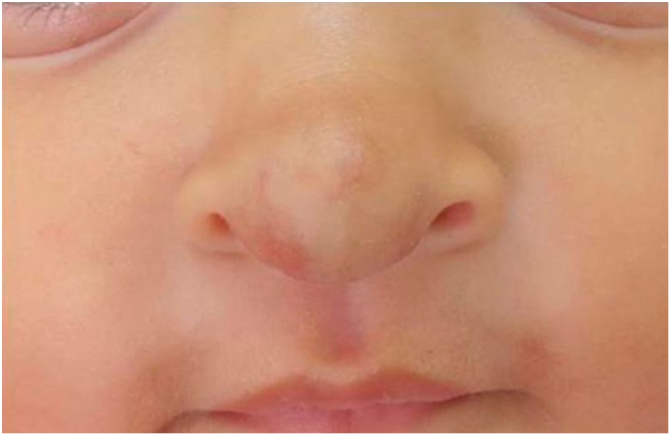
After the treatment with oral propranolol, 9-month-old infant.

**Figure 4:**
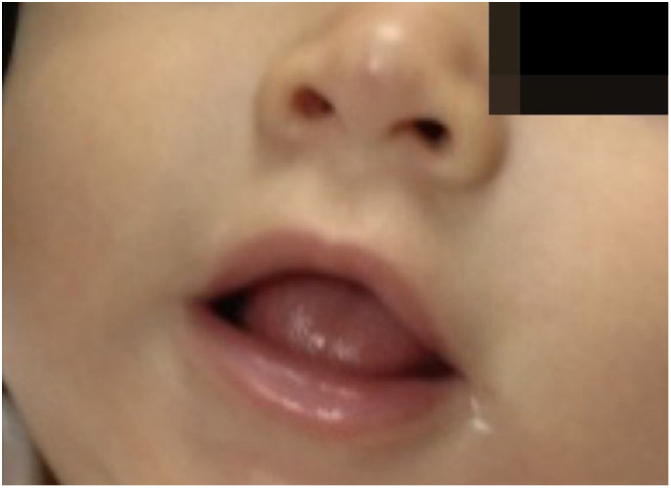
After the treatment with oral propranolol, 5-month-old infant.

The infants developed none of the reported side effects – sleep disturbances, hypoglycemia or hypotension – and achieved total involution without deformities or residuals scars, demonstrating the efficacy of oral propranolol and its advantages in relation to corticosteroids. The patients did not present recurrence in the 3 months that followed (Figure 3, Figure 4).

## Discussion

In view of possible complications, effective treatment without side effects is necessary. Formerly, for IH located in the nasal tip, systemic corticosteroids, laser therapy and surgical excision were the most common therapeutic options to delay or interrupt the growth of the vascular tumor and enable its removal. High doses of corticosteroids during prolonged periods are frequently accompanied by several side effects, particularly in children, such as decreases in bone formation and increases in its absorption, proximal weakness and reduction of bactericidal activity, facilitating infections. In addition, consequences can include increased hepatic gluconeogenesis, increased insulin resistance, psychosis and mood changes. Equally, laser therapy and surgical removal can lead to unesthetic scars.^4^, ^5^, ^6^

Hence, the use of effective drugs with fewer side effects is paramount. Beta-blockers, specifically propranolol, therefore became the first line of therapy and were approved by the Food and Drug Administration in March 2014. However the use in Brazil is not yet widespread.^6^, ^7^

Beta-Blockers (BB) are beta-adrenergic antagonists that generate systemic actions, including reduction of cardiac output through a decrease in sinoatrial node activity. This provokes hypotension, causing reflex peripheral vasoconstriction, which is believed to be responsible for involution of the hemangioma. Furthermore, BBs can generate bronchoconstriction, increases in sodium retention and plasma volume secondary to the reduction in renal perfusion, as well as decreased glycogenolysis and glucagon secretion, predisposing to hypoglycemia. Specifically concerning the treatment of IH, BBs have been linked to hypoglycemia, nightmares and hypotension.^3^, ^6^, ^8^

Regarding the risks involved, it is necessary to perform cardiologic and pulmonary evaluation before treatment. Initially, an Electrocardiogram (ECG) must be requested and Blood Pressure (BP), Cardiac Frequency (CF) must be checked, combined with detailed cardiologic and pulmonary physical examination. At the beginning of treatment (the first dose or upon an increase in the dose) the following should be checked every hour during the 4 h period: temperature, CF, respiratory rate and pulmonary auscultation. Many authors recommend that ECG and capillary glycemia must also be performed prior to administration, and 120 and 240 min thereafter, thus avoiding the risk of hypotension, bronchospasm and hypoglycemia.^3^, ^8^, ^9^

Compared to corticosteroids, BBs induce a faster response, with a response rate of around 90%. Moreover, there is a lower risk of relapse and a decreased need for surgical intervention.^3^, ^8^, ^10^ In our experience, propranolol has resulted in excellent therapeutic response and safety, as exemplified by the two aforementioned cases.

## Financial support

None declared.

## Authors’s contributions

Mariana Carvalho Costa: Approval of the final version of the manuscript; conception and planning of the study; elaboration and writing of the manuscript; obtaining, analysis, and interpretation of the data; effective participation in research orientation; intellectual participation in the propaedeutic and/or therapeutic conduct of the studied cases; critical review of the literature; critical review of the manuscript.

Odil Garrido Campos de Andrade: Approval of the final version of the manuscript; conception and planning of the study; elaboration and writing of the manuscript; obtaining, analysis, and interpretation of the data; critical review of the literature; critical review of the manuscript.

Lethícia de Castro Pereira: Approval of the final version of the manuscript; elaboration and writing of the manuscript; critical review of the manuscript.

Izelda Maria Carvalho Costa: Approval of the final version of the manuscript; elaboration and writing of the manuscript; obtaining, analysis, and interpretation of the data; effective participation in research orientation; intellectual participation in the propaedeutic and/or therapeutic conduct of the studied cases; critical review of the literature; critical review of the manuscript.

## Conflicts of interest

None declared.
